# Penile and Scrotal Strangulation due to Metal Rings: Case Reports and a Review of the Literature

**DOI:** 10.1155/2018/5216826

**Published:** 2018-03-27

**Authors:** Neel H. Patel, Ariel Schulman, Jonathan Bloom, Nikil Uppaluri, Michael Iorga, Suraj Parikh, John Phillips, Muhammad Choudhury

**Affiliations:** ^1^Department of Urology, New York Medical College, Valhalla, NY, USA; ^2^Department of Urology, Duke University Medical Center, Durham, NC, USA; ^3^Urologic Oncology Branch, National Cancer Institute, Bethesda, MD, USA

## Abstract

Penile and scrotal entrapment from a metal ring placed at the base of the penis is a rare, but important clinical dilemma encountered in urology. Emergent presentation to the urologist, after ring placement far longer than safely practiced, risks ischemic and permanent injury to penile, scrotal, and intrascrotal structures. Treating urologists should be aware of the prevalence of metal ring use, their potential complications, and the surgical approach to their safe removal. We present two patients who were identified at our institution with strangulating injuries of retained penile rings. The first patient was a healthy, 43-year-old male with a metal ring retained for 24 hours that was safely removed with industrial bolt cutters. The second patient, a 74-year-old male, died as a result of sepsis from injuries secondary to penoscrotal ischemia after >48 hour ring retention despite prompt removal at emergent presentation. Although rare, sexual practices may include the use of penoscrotal rings. When retained, ischemic injury and edema may lead to strangulation. Emergent removal may require industrial equipment that is not within the confines of normal operating room tools. Tissue injury may be severe and sepsis life-threatening, even after ring removal.

## 1. Introduction

Penile ring strangulation as well as concomitant scrotal entrapment can present as a challenging urological emergency. Metal rings in theory increase penoscrotal engorgement during sexual activity. Detumescence may facilitate removal while nonremoval may lead to delayed detumescence, edema, and then the cycle of tissue injury with ischemia and necrosis [1]. Of these presentations, constricting ring injury may be more commonly associated with ring materials of plastic, Teflon, or rubber, which are more amenable to surgical removal. Some penile rings are composed of titanium/metallic alloy, of heavy density, and can withstand common management tactics [2]. Various techniques have been described for removing constricting devices including lubricants, coiled strings/gauze, needle aspiration, and cutting of the ring itself [2–5]. Here we report our approach to two cases of penile strangulation with different clinical presentations requiring surgical treatment.

## 2. Case Presentation

### 2.1. Case 1

**Figure 1:**
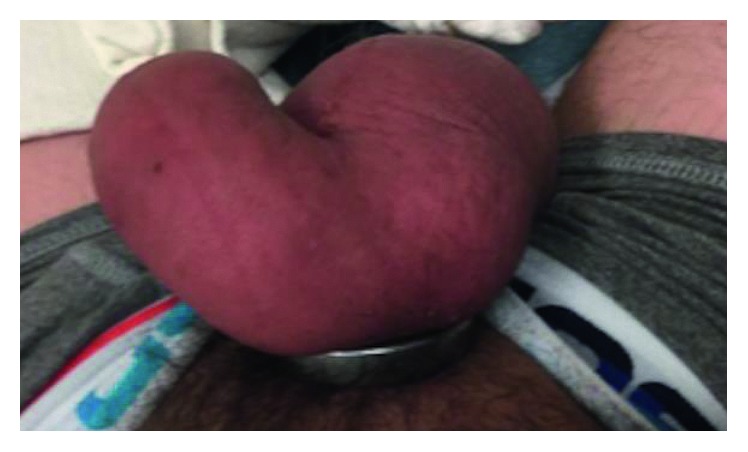
Case 1: metal ring encircling phallus and scrotum.

A 43-year-old man with no significant medical or psychiatric history presented to our emergency room (ED) with a 24-hour history of strangulated penis. The patient had placed both his phallus and scrotum through a metallic ring for sexual enhancement and then was unable to remove the ring after intercourse. The ring measured 6 cm in diameter and it was 1 cm thick. The patient complained of lower abdominal pain and decreased sensation to his genitalia. On physical exam, the patient was found to have severe swelling of his penis and scrotum distal to the ring, which was placed at the base of these structures as seen in Figure 1. There remained approx. 1 cm of space between ring and edematous genitals, but no possibility of manual removal. No necrotic tissue could be appreciated.

**Figure 2:**
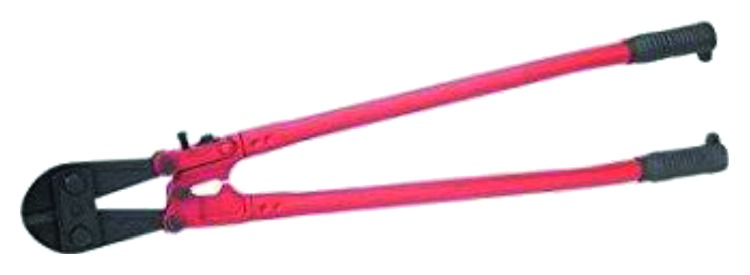
Industrial grade steel bolt cutters.

Attempts by ED staff to remove the ring using lubrication and the finger ring-cutter were unsuccessful. Urology was then consulted, and the patient was taken to the Operating Room (OR) to receive general anesthesia to allow for more invasive removal options. Further attempts were made in the OR using an orthopedic pin cutter and gigli saw, which were limited. A handheld rotating electric saw was used and appeared to make progress; however, a high amount of heat was transmitted around the ring causing a first degree circumferential burn injury that could not be prevented despite use of irrigation during sawing to keep the ring cool. Industrial bolt cutters (Figure 2) were obtained from the maintenance department and were used to cut the ring at the 12 and 6 o'clock position, allowing for removal of the ring. Penile detumescence was achieved within the next hour, and the patient was discharged the following evening with oral antibiotics and pain control. One-week follow-up revealed that the patient had full recovery with good urinary and erectile function.

### 2.2. Case 2

**Figure 3:**
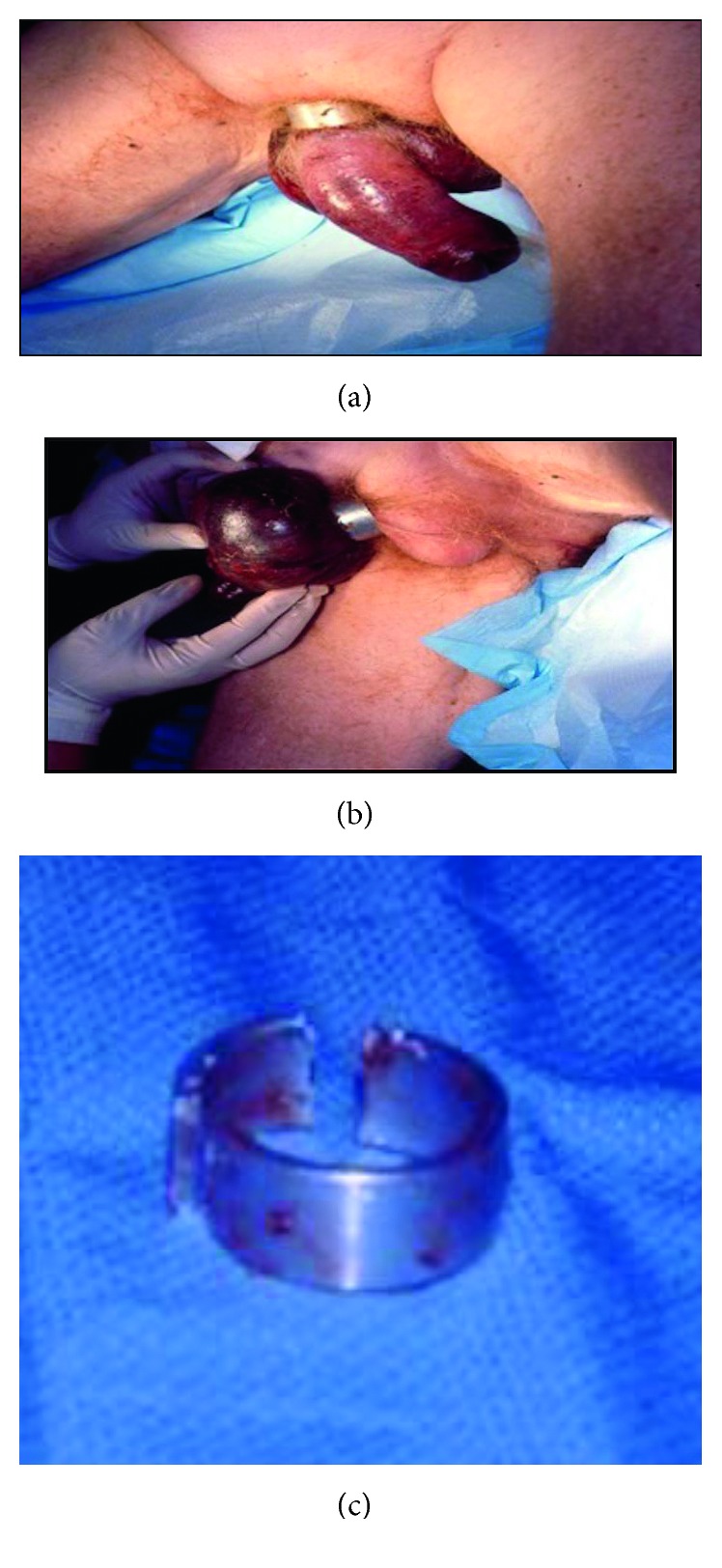
(a) Case 2: metal ring encircling phallus and scrotum. (b) Areas of significant necrosis seen along scrotum. (c) Metal ring disassembled with bolt cutters.

A 74-year-old man presented to our ED with placement of a metal penoscrotal ring for over 48 hours. The patient had a history of multiple medical comorbidities including cirrhosis and diabetes mellitus. The patient was found by family and presented to the hospital in an obtunded state, with fevers, and an elevated white count. On physical exam, the patient was found to have ring placement at the base of his penis and scrotum. Severe swelling of the affected area was seen as shown in Figure 3(a). Due to the prolonged onset of presentation (>48 hours), necrosis of the scrotum could be seen as well, as demonstrated in Figure 3(b). The patient was taken emergently to the OR and had removal of the ring with the use of bolt cutters with cuts in two separate parts allowing for removal of the device (Figure 3(c)). Despite aggressive resuscitation in the intensive care unit, the patient continued to be in septic shock postoperatively and died due to his condition.

## 3. Discussion

Penile strangulation presents as a urologic emergency usually brought on by the patient for enhancement of sexual function. Presentation is often likely delayed due to embarrassment. Treatment requires prompt removal of the constricting device to allow for return of blood flow and relief of urinary obstruction. Early success with removal of the constricting ring will limit the ischemia time and subsequent sequelae of necrosis and loss of function, that is, erectile and urinary function.

A five-stage grading system was developed by Bhat et al. to also help characterize these injuries [4]. The spectrum of severity on the Bhat scale ranged from Grade I causing edema of the distal penis, to Grade V presenting with gangrene, necrosis, or complete amputation (Table 1). Approaches to removal of constricting devices have a wide range of options, which can depend on the device composition and size, as well as degree of edema/strangulation. There have been reports demonstrating use of needle aspiration, electric tools, saws, industrial bolt cutters, and assistance from firemen and maintenance staff (Table 2) [1, 2, 6–12].

Our case presentations demonstrate multiple tactics progressing from simple emergency room options to a variety of tools that required use in a controlled setting within the operating room. We suggest the involvement of other individuals such as orthopedic or trauma surgery, the maintenance department, or even emergency or fire personnel for their knowledge of tools foreign to most urologists. Mechanical methods of device removal should be preferred over electrical/thermal devices to reduce the possibility of burn injury as well as urethrocutaneous fistulas or urethral strictures [10]. The device should be cut in two places, ideally 180 degrees apart for easy removal. If electrical tools are required, then care must be taken to protect the underlying and adjacent tissue, as well as cool the device while cutting. Patients with extensive medical comorbidities and those at high risk for postoperative complications should be monitored appropriately and have adequate follow-up.

Our second case presentation demonstrates the possibility of death from penile strangulation due to concomitant sepsis and multiple medical comorbidities. To our knowledge, this is the second such reported incident. Morentin et al. initially presented death as a result of multiorgan failure due to septic shock in a patient with penile strangulation and multiple medical comorbidities from a plastic bottle for approximately two weeks [11].

## 4. Conclusion

Penile strangulation presents as a urological emergency, and if not managed in a timely fashion, it can lead to ischemic complications such as necrotic tissue or wounds or sexual and urinary dysfunction. Prolonged episodes in patients with multiple medical comorbidities can even result in death. Multiple methods of management have been presented in the literature. Tools unfamiliar to the surgeon and the assistance of other departments in an institution may be needed for prompt management and reduction of the strangulation. Mechanical methods of removal may be preferred to avoid any injury from thermal/electrical burns.

## Figures and Tables

**Table 1:** Penile strangulation classification system by Bhat et al.

Grade I	Distal penis edema. No evidence of skin ulceration or urethral injury.
Grade II	Distal penile edema with decreased sensation. Injury to skin, constriction of corpus spongiosum. No urethral injury.
Grade III	Injury to skin and urethra, without urethral fistula. Loss of distal penile sensation.
Grade IV	Complete division of corpus spongiosum leading to urethral fistula and constriction of corpus cavernosum with loss of distal penile sensation.
Grade V	Gangrene, necrosis, or complete amputation of penis.

**Table 2:** Presentation of various case reports with penile strangulation injury.

Review of reported cases						
	Time to presentation	Comorbidities	Device	Penile condition	Removal technique	Long-term sequelae
Chennamsetty et al. [7]	9 days	None	7 mm thick, alloy ring	Skin necrosis	Orthopedic pin cutter	None
Singh et al. [1]	26 hours	None	Metallic ball bearing ring	Edema/discoloration	Needle aspiration/manual decompression	None
Talib et al. [2]	8 hours	Erectile dysfunction	2.5 × 1.5 cm metallic ring	Penile edema/congestion	Rotating saw	None
	6 hours	None	1 cm thick metal ball bearing ring	Penile edema	4 needle aspiration	None
Santucci et al. [6]	72 hours	Schizophrenia	10 lb barbell	Penile edema/discoloration	Air grinder saw	None
Eaton et al. [8]	16 hours	None	1 cm thick × 2 cm wide × 6 cm diameter ring	Penile edema/hyperemia	Gigli saw	None
Huang et al. [9]	—	Diabetes mellitus, coronary artery disease	Plastic bottle	Penile edema	Dental drill	None
Zhang et al. [12]	2 days	None	3 cm diameter × 2 mm thick metallic ring	Penoscrotal edema	Hydraulic cable cutter	None
Kyei et al. [10]	12 hours	None	2 cm wide × 0.8 cm thick metallic nut	Penile edema	Bosch electric grinder	Thermal injury—urethrocutaneous fistula and urethral stricture
Morentin et al. [11]	2 weeks	Cerebral vascular accident, smoking, alcoholism, social behavior disorder	Plastic bottle	Necrosis/gangrene	None	Death—multiorgan failure due to sepsis
